# Validating reference-based algorithms to determine cell-type heterogeneity in ovarian cancer DNA methylation studies

**DOI:** 10.1038/s41598-024-61857-y

**Published:** 2024-05-14

**Authors:** Edyta Biskup, Joanna Lopacinska-Jørgensen, Lau Kræsing Vestergaard, Estrid Høgdall

**Affiliations:** Department of Pathology, Copenhagen University Hospital, Herlev, Denmark

**Keywords:** Reference-based deconvolution, Ovarian cancer, DNA methylation, Tissue heterogeneity, Cancer microenvironment, Tumour heterogeneity, Computational biology and bioinformatics, Epigenetics

## Abstract

Information about cell composition in tissue samples is crucial for biomarker discovery and prognosis. Specifically, cancer tissue samples present challenges in deconvolution studies due to mutations and genetic rearrangements. Here, we optimized a robust, DNA methylation-based protocol, to be used for deconvolution of ovarian cancer samples. We compared several state-of-the-art methods (HEpiDISH, MethylCIBERSORT and ARIC) and validated the proposed protocol in an in-silico mixture and in an external dataset containing samples from ovarian cancer patients and controls. The deconvolution protocol we eventually implemented is based on MethylCIBERSORT. Comparing deconvolution methods, we paid close attention to the role of a reference panel. We postulate that a possibly high number of samples (in our case: 247) should be used when building a reference panel to ensure robustness and to compensate for biological and technical variation between samples. Subsequently, we tested the performance of the validated protocol in our own study cohort, consisting of 72 patients with malignant and benign ovarian disease as well as in five external cohorts. In conclusion, we refined and validated a reference-based algorithm to determine cell type composition of ovarian cancer tissue samples to be used in cancer biology studies in larger cohorts.

## Introduction

The heterogeneity of tumor tissue is an important aspect to consider in biomarker discovery. It is widely acknowledged that tumors are not simple aggregates of cancer cells but constitute complex microenvironments. Their composition may vary between tumor types, but overall they consist of cancer, stromal and immune cells, which interact either indirectly, by secreting signaling molecules, or directly, through membrane receptors^1,2^.

Tumor heterogeneity is a technical challenge as it may impact reproducibility, but it can also play a role in biomarker studies helping to predict response to treatment and prognosis. For example, M2-type tumor associated macrophages (TAMs) typically correlate with advanced stages and poor clinical outcomes in ovarian cancer (OC)^3,4^. In line with these observations, Peng et al. suggested that the TAMs profile may be useful for predicting patients’ response to immune- and chemotherapy^5^. Apart from immune cells, cancer-associated fibroblasts (CAFs) and endothelial cells (EC) may also contribute to tumor development either directly influencing proliferation, invasion, and migration of cancer cells (CAFs) or via angiogenesis^2^.

On the other hand, intra-sample heterogeneity may be a confounding factor. Hence, it either masks biological findings or creates false positives. Therefore, when performing gene expression or methylation analysis, results should be corrected for cell proportions to ensure consistent results. Otherwise, it remains unclear whether the observed differences are due to the condition in question (for example cancer versus healthy control) or due to sample cell composition^6,7^.

Sample cell type composition can be established either by experimental techniques (such as flow cytometry and single-cell RNA-Seq) or by computational methods that are designed to deconvolve (“unmix”) signals from bulk tissue samples. The latter is less costly and less technologically demanding as it does not require fresh tissue samples. Specifically, such tissue samples have to be gently disaggregated, to avoid cell lysis, and this step is often not feasible, hampering the process^8^.

Either gene expression or methylation profiles can be used as input data for deconvolution. Deconvolution protocols using expression data (mRNA-Seq) were implemented earlier and gained a broader use, with 20 deconvolution protocols based on mRNA-Seq data being available up to date^9^. Deconvolution based on DNA methylation (DNAm) profiles is somewhat less studied, with the first protocol proposed by Houseman in 2012^10^. Overall, they can be divided into reference-free and referenced-based algorithms with the latter shown to be more precise^11^. Reference-based algorithms require information in the form of reference profiles. Those are generated from experimental data, such as single-cell RNA sequencing (scRNA-seq) or mRNA-Seq of isolated cell populations and can be derived from publicly available repositories, such as Gene Expression Omnibus.

Using methylation profiles as input for tissue deconvolution has several advantages as compared to using mRNA-Seq. First, methylation markers are stable and cell specific. DNA itself is more stable than RNA and methylation is a binary event, rather than a gradual change like gene expression level, so it can be tracked with the cell number. Lastly, the number of features (i.e. potential methylation sites versus genes expressed) is several orders of magnitude higher as there are approximately 28.3 × 10^6^ CpG sites in the human genome compared to approximately 20,000 genes^7,12^.

OC is an aggressive malignancy, originating in the female genital tract. Its symptoms are usually discrete and nonspecific, which delays the diagnosis and significantly worsens patients’ prognosis^13^. OC may arise in the ovary itself or from neighboring structures, most notably in the distal fallopian tube^14^. Epithelial OC is a heterogeneous disease with serous adenocarcinoma being the most common histological subtype, followed by mucinous-, endometrioid adenocarcinoma and clear cell carcinoma, and other less common “unspecified” epithelial subtypes^15^. Each histological OC subtype differ with respect to mutagenic profile, and methylation patterns^16^.

The aim of our study, rather than systematically evaluate existing protocols, was to identify a reliable and robust method, which could be applied to estimate cell proportions in OC tissue samples, using DNAm profiles. This information is suitable for use in exploratory studies on OC biology and potentially to support/assist in treatment guidance. We also believe that similar considerations may be relevant when designing reference panels for other types of cancers. In the current study we only included reference-based protocols which were previously systematically compared to other protocols and provided reliable cell proportions estimates.

## Materials and methods

### Patients and tissue samples

Our study cohort consisted of tissue samples from 72 patients admitted to Gynecologic Clinic at Rigshospitalet (Copenhagen, Denmark) included in the Pelvic Mass/GOVEC study. Patients were diagnosed as 11 benign cases, 8 with borderline ovarian tumors, 37 with high grade serous ovarian carcinoma (HGSOC), 8 with other histological subtypes of OC (OC-other) and 8 cases diagnosed with primary cancer other than OC (not-OC). All patients were Caucasians. Information of patients’ age and disease stage can be found in Table 1.

**Table 1:** Clinicopathological features. Data was retrieved from The Danish Gynecological Cancer Database (DGCD; www.dgcg.dk/) register. *OC-other* OC subtypes other than HGSOC, *not-OC* malignant mass present in ovaries, but with primary cancer not being OC, *NA* stage not specified.

	Benign	Borderline	HGSOC	OC-other	Not-OC
No. of cases	N = 11	N = 8	N = 37	N = 8	N = 8
Median age in years (range)	57.5 (20.5–77.4)	59.8 (39.0–86.2)	65.7 (41.1–84.1)	60.7 (31.9–83.1)	65.5 (52.9– 83.4)
FIGO Stage					
I		4	1		1
II		1	6	2	2
III		1	30	3	1
IV		2		2	1
NA				1	3

Fresh frozen tissue samples were cut into small pieces (2–3 mm) with a sterile scalpel and homogenized in TE buffer, prior to DNA extraction with the Maxwell^®^ RSC Tissue DNA Kit (Promega, Madison, WI) and a purifications step with the QIAquick PCR-purification system (Qiagen, Hilden, DE). DNA samples were quantified using the Qubit fluorometer (Thermo Scientific, Waltham, MA) and subsequently bisulfite-converted (500 ng per sample) using EZ DNA methylation kit (Zymo Research, Irvine, CA). The global DNA methylation was profiled using Infinium^®^ MethylationEPIC BeadChip Kit according to manufacturer’s instructions (Illumina, San Diego, CA). Raw data was stored as .idat files and processed as described below (see “Data acquisition and processing of DNA methylation profiles”).

### External datasets

Following datasets, containing ovarian cancer samples and cancer-free normal controls were used for method validationGSE133556^17^, GSE51820, GSE72021^18^, GSE155760^19^, and GSE168930^20^. Furthermore, other datasets (Table 2), containing methylation profiles of pure cells lines (primary or cancer) and/or purified blood cells were used for construction of reference panels.

**Table 2:** List of datasets used in the current work for constructing reference panels. If a given dataset contained more samples of the same cell type, only samples with untreated/non-transfected cells were used.

Type of sub-panel	GEO number	Content	Number of samples
Non-neoplastic cells HEpiDiSH ref panel—set A	GSE35069^21^	Immune cells	
		CD4T	N = 6
		CD8T	N = 6
		B cells	N = 6
		Monocytes	N = 6
		NK cells	N = 6
		Neutrophils	N = 6
		Eosinophils	N = 6
	GSE40699^22^	Epithelial cell lines	N = 11
		Fibroblast cell lines	N = 7
Non-neoplastic cells Independent ref panel—set B	GSE110555^23^	Immune cells	
		CD4T	N = 7
		CD8T	N = 6
		B cells	N = 6
		Monocytes	N = 6
		NK cells	N = 6
		Neutrophils	N = 6
	GSE88824^24^	Immune cells	
		CD4T	N = 8
		CD8T	N = 8
		B cells	N = 8
		Monocytes	N = 8
		NK cells	N = 8
		Neutrophils	N = 8
	GSE49618^25^	Immune cells	
		B cells	N = 3
		Monocytes	N = 3
	GSE74877^26^	Epithelial cells	N = 2
		Fibroblasts	N = 4
	GSE52074^27^	Epithelial cells	N = 3
	GSE54758	Epithelial cells	N = 3
	GSE111505	Fibroblasts	N = 1
Cancer component—set C	GSE68379^28^	Ovarian cancer cell lines	N = 43
Cancer component—set D	GSE168225^29^	Ovarian cancer cell lines	N = 4
	GSE40699^22^	Ovarian cancer cell lines	N = 1
	GSE56621^30^	Ovarian cancer cell lines	N = 3
	GSE57342^31^	Ovarian cancer cell lines	N = 26
	GSE198073^32^	Ovarian cancer cell lines	N = 6

### Data acquisition and processing of DNAm profiles

For samples from the study cohort and for samples from external datasets where the raw data (.idat files) were available, those were parsed into R using minfi package^33^ and processed as described by^34^. Normalization was conducted using preprocessFunnorm^35^. Quality assessment was based on the median signal intensity of both methylated and unmethylated probes^33^. Samples with median log_2_ values below 10.5 were discarded due to low quality (two samples from the study cohort: one benign ovarian disease and one borderline ovarian tumor sample). Each main cell type (fibroblasts, epithelial, cancer and immune cells) was processed separately. If both 450 K and EPIC arrays were available for the same cell type, a combineArrays() function was used and only the CpGs shared between 450 K and EPIC platforms were kept.

If the raw files were not available, min–max normalization of the beta values was performed.

Cross-reactive probes and probes affected by SNPs were filtered out in all datasets. Additionally, probes localized on sex chromosomes were filtered out in datasets used for construction of reference panel. This was done because the gender of the reference samples did not always match the study cohort and the reference cohort (GSE133556), and thus they could not have been used for deconvolution. In the study cohort probes localized on sex chromosomes were kept in order to be used in future studies, as they may be biologically relevant.

**Figure 1:**
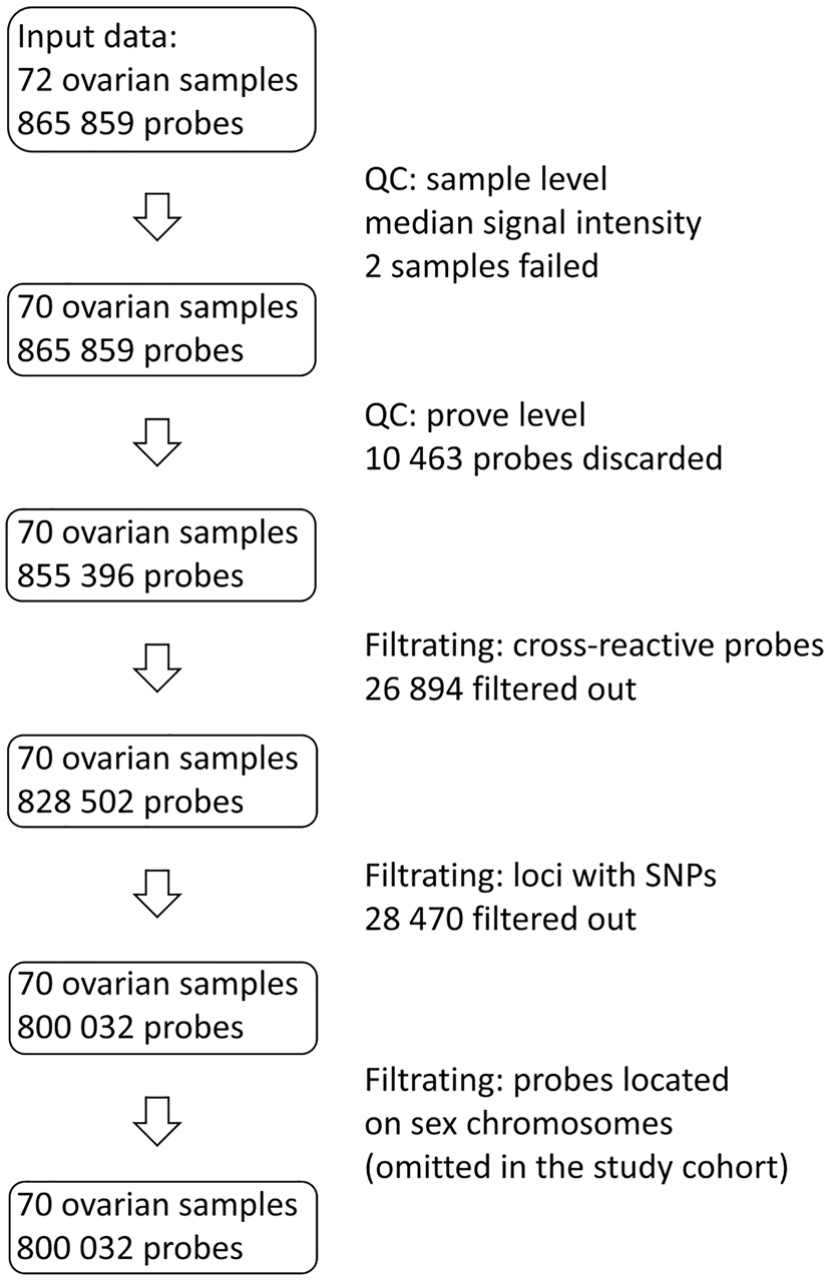
Workflow illustrating data curation for the study cohort. First quality control (QC; on the sample level) is performed basing on median signal intensity of methylated and unmethylated probes as described in Ref.^33^. QC on the probe level, filtering out cross reactive probes and probes affected by SNPs as described in Ref.^34^.

A simplified workflow for the study cohort, together with the outcome of each filtration step, is shown in the Fig. 1.

### Deconvolution protocols used

The following reference-based protocols were used for estimating cell-type proportions: HEpiDiSH^36^, MethylCIBERSORT^8^ and ARIC^37^ For the comparison of main differences between protocols used, refer to Table 3. Moreover, details related to how each of the protocols has been implemented here are gathered in Supplementary Table S1.

**Table 3:** Overview of the deconvolution protocols used.

Name	Platform based	Strategy of CpG selection	Computational algorithm	Source
HEpiDISH	R	One-against-all	Robust partial correlations (RPC)	^36^
MethylCIBERSORT	R/online platform	Pairwise	Support vector regression (SVR)	^8^
ARIC	Python	Pairwise	Weighted υ-SVR	^37^

### HEpiDISH

HEpiDISH is available as an R-based script and allows quick estimation of the amount of epithelial, fibroblasts and immune cells in epithelial samples, using a predefined reference panel (built using datasets from set A, Table 2). It works as a stepwise deconvolution—first, it provides information about the three main underlying cell types, second, it breaks the immune component into seven immune cell subtypes^36^.

Nomeenclature used: “EpiDISH” refers to the basic name of the deconvolution procedure, allowing to estimate proportions of three main cell types: epithelial (Epi), fibroblasts (Fib) and immune cells (IC). “HEpiDISH” refers to “hierarchical “EpiDISH”, a modification allowing further resolution of the immune component into seven cell subtypes . Here, we use the term “HEpiDISH” throughout the whole article.

Apart from coming with a predefined reference panel, HEpiDISH also has an option to apply a user-defined reference panel.

### Adding information about tumor component to the HEpiDISH reference panel

In order to identify proportion of tumor cells in OC samples, we modified a deconvolution protocol previously described in^36^ by adding CpGs specific for the tumor component (TC) to their reference panel. When constructing the modified panel, for the three main cell categories: “Epi”, “Fib” and “IC” we used cell lines and purified blood cells previously used by^36^ and originally generated as a part of ENCODE project^22^ (Epi and Fib) or published by^21^ (immune cells) (Table 2, set A). For the tumor component (TC) category we used 43 OC cell lines from^28^ (Table 2, set C).

As in the original protocol, we used the “one-against-all” strategy, i.e., we searched for CpGs characteristic for each group (for example for tumor component; TC) by comparing this group with the remaining three (for example TC versus Epi + Fib + IC). We demanded, apart from FDR < 0.01, also the following distances between average methylation levels of at least: 0.85 between IC and the rest, 0.65 between Fib and the rest, 0.55 between Epi and the rest and 0.8 between TC and the rest. These values ensured identification of 150–300 CpGs for each comparison (specifically IC vs all: resulted in 171 CpGs, Fib vs all: 200 CpGs, Epi vs all: 152 CpGs, and TC vs all 228 CpGs). Finally, we identified 746 unique CpGs, which would allow performing the first step of deconvolution, i.e., identification of four main cell types (Supplementary Table S2).

To perform the subsequent step (prediction of immune cell subtypes), Zheng et al., identified 188 CpGs, which did not overlap with those meant for identification of the three main cell types^36^. Those we also kept unaltered, i.e., we did not perform a new selection. However, due to our filtration criteria (see, “Data acquisition and processing of DNA methylation profiles”) their number was reduced from 188 to 169. Hence, in total our updated panel contained 746 + 169 = 915 CpGs.

### Details on application of the other deconvolution protocols

MethylCIBERSORT is a flexible and versatile deconvolution tool. It includes an R-based interface for feature selection (CpG sites), allowing users to decide which cell types to expect and which datasets to include in reference panel construction^8^. We based our reference panels on datasets gathered in Table 2 in the following combinations: A + C, B + D and A + B + C + D, where A + C corresponds to the reference panel used by HEpiDISH-OC, B + D is a reference panel independent from the one used by HEpiDISH-OC and A + B + C + D is a broad reference panel, containing all 247 datasets, both for normal and malignant cells (see also Supplementary Table S1).

Reference panels were constructed with a limma-based function for feature selection (FeatureSelect.V4) built in MethylCIBERSORT package with default settings (i.e., allowing maximum 100 features per pairwise comparison, median β-value difference of 0.2 and FDR < 0.01)^8^.

Custom reference panel obtained this way was then uploaded to https://cibersortx.stanford.edu/ website and deconvolution was performed online, using CIBERSORTx platform, setting the number of iterations to 500 (see Supplementary Table S1). The R markdown file, showing implementation of MethylCIBERSORT, as well as the reference panel based on A + B + C + D sets, can be found in the Supplementary Data folder. A tab-delimited reference panel (Normal_and_OC_ref_ABCD_Signature.txt) can be used alongside a beta-values matrix provided by a user and uploaded directly to CIBERSTORTx platform.

ARIC is a Python-based method. Motivation behind its design was a need for an accurate deconvolution of rare cell types. It accepts a list of preselected CpG sites (in our case: about 15,000; see Supplementary Table S1), and then performs a further feature selection, aiming to minimize collinearity between methylation profiles of given cell types, followed by the actual deconvolution^37^. Pre-selection is necessary, since using a full methylation profile would be too computationally heavy. In our case, for pre-section we used the feature selection function (FeatureSelect.V4), built in MethylCIBERSORT, however, but we allowed maximally 1000 instead of default 100 CpGs (Supplementary Table S1).

An important difference between HEpiDISH versus MethylCIBERSORT and ARIC is that the latter two do not contain a designated step for resolving immune component but perform deconvolution of all cell types simultaneously. Each utilizes a different computation algorithm for deconvolution (Table 3).

### Construction of the synthetic datasets

Accuracy of the estimates of the tested deconvolution protocols were verified using a synthetic dataset. Briefly, we prepared an in silico set of bulk samples with known proportions as follows.

The Dirichlet probability distribution was applied to generate cell compositions. We performed the simulation twice, each time for 10,000 synthetic mixtures. First, we allowed nine cell types in each synthetic sample, then ten cell types in varying proportions. First set of the synthetic samples (allowing nine cell types) was used to evaluate the effect of unbalanced cell type proportions on performance of HEpiDISH. Second one was used to compare performance of seven deconvolution protocols.

This data generation was conducted in Python (v.3.9.18) using the NumPy package (v.1.26.0) with the built-in function dirichlet.

**Figure 2:**
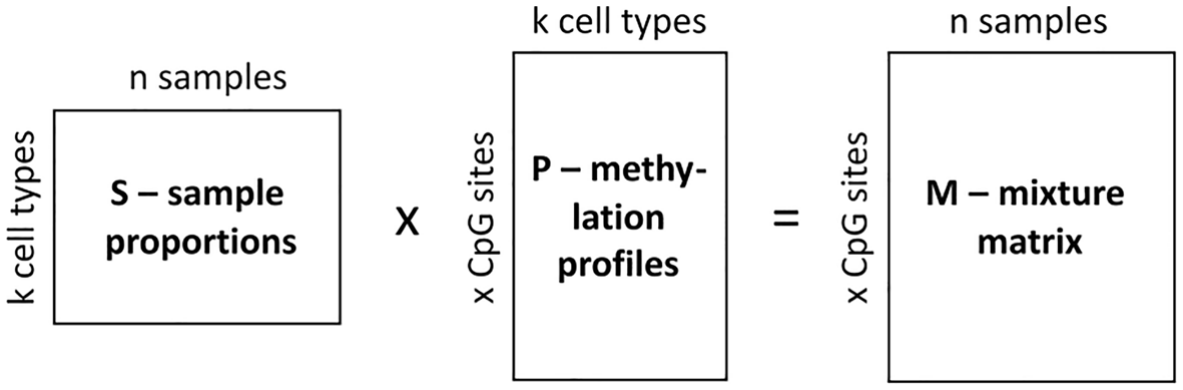
Simplified model of synthetic bulk samples preparation. *S* matrix sample proportions, *P* matrix of methylation profiles, specific for underlying cell types, *M* mixture matrix, i.e. matrix containing information about methylation profiles of synthetic bulk samples.

A matrix representing this set of sample proportions (**S**; Fig. 2) was then multiplied by another matrix, containing methylation profiles (**P**), specific for underlying cell types. A resulting, third matrix contained beta values of the synthetic bulk samples (mixture matrix; **M**), which is linear combination of methylation profiles of different cell types. Deconvolution algorithm, if performing correctly, should be able to reconstruct the **S** matrix, when matrix **M** and a suitable reference panel are used as input data.

Methylation profiles (matrix **P**) were prepared by averaging beta-values of randomly selected samples from sets A, B, C and D (Table 2). For non-neoplastic cells, we selected four samples from each cell type (two from A and two from B, apart from eosinophils which were absent in set B) and 12 for tumor cells (six from set C and six from set D). Altogether, we prepared three methylation profile datasets, and each sample from matrix **S** was multiplied by all three, generating 20 × 3 = 60 synthetic samples. We reasoned that since methylation profiles even for the same cell type would differ (due to inter-lab variation or intrinsic characteristic of a subject), the bulk synthetic dataset should not be built based on only one methylation profile. At the same time, we made sure that methylation profiles used for construction of matrix **S** differed from reference panels.

Cell types we included in construction on the synthetic datasets were as follows: epithelial, fibroblasts, B lymphocytes, CD4 + and CD8 + lymphocytes, natural killer cells, monocytes, neutrophils, eosinophils, and ovarian cancer cells. In some cases, we restricted the total content of immune cells, so that the whole immune content did not exceed a desired value. This was done to see the effect of unbalanced cell proportions on the deconvolution accuracy (see “Results”).

### Evaluation of deconvolution accuracy

To verify if the deconvolution results are accurate, we used the following metrics: Spearman correlation between actual cell proportions and cell proportions predicted by the model, root mean square error (RMSE) and median absolute percentage error (MedAPE).

Spearman correlation was chosen following normality check, performed by Shapiro–Wilk method.

RMSE gives an information about the overall accuracy of the deconvolution model. Meanwhile, MedAPE is adjusted for the relative content of a given cell type. Mean absolute percentage error (MAPE) was adopted by^37^ who used it to check for accuracy of estimation of rare cell types. Cell types present in low amounts contribute less to the overall RMSE, however, for a given rare cell type the error might be substantial. In our study we decided to implement median rather than mean, as we observed that the errors (differences between actual and predicted cell proportions for a given cell type) were not distributed normally. Thus, by using median we made sure that a single cell type in a single sample would not unproportionally inflate the error. Simplified notations of both metrics are as follows:

$$ {\text{RMSE }} = {\text{ sqrt}}\left( {{\text{mean}}\left( {{\text{p}}_{{\text{m}}} {-}{\text{ p}}_{{\text{a}}} } \right)^{{2}} } \right) $$

$$ {\text{MedAPE }} = {\text{ median}}\left( {{\text{abs}}\left( {\left( {{\text{p}}_{{\text{m}}} {-}{\text{ p}}_{{\text{a}}} } \right)/{\text{p}}_{{\text{a}}} } \right)} \right) $$

where p_m_ is the cell proportions predicted by the model; p_a_ is the actual cell proportions.

Moreover, when constructing HEpiDISH-OC, we also performed a sanity check as suggested by^38^. Briefly, we verified how the protocol would recognize datasets used for construction of its own reference panel (sets A and C, “recognizing-self”). We also tested how HEpiDISH-OC would recognize independent datasets, i.e., datasets not included in the HEpiDISH-OC reference panel (sets B and D, “recognizing-other”).

### Data analysis

Statistical analysis (including correlations and dimensional reduction) as well as graph preparation were performed in R programming language (version 4.2).

### Ethics approval

The study was carried out according to the guidelines of the Declaration of Helsinki, including written informed consent from all subjects, and it has been approved by the Danish National Committee for Research Ethics, Capital Region (approval codes KF01-227/03 and KF01-143/04). All patients were followed until either death of any cause, emigration or January 17th, 2015.

### Consent to participate

Informed consent was obtained from all individual participants included in the study.

## Results

### Defining a range of cell type proportions

A significant part of our work was performed using artificial methylation datasets, to allow comparison of the deconvolution results with the ground truth. Since we did not expect the proportions of cell types to be equal, we first evaluated how low-abundance cell types would affect the reliability of deconvolution. To do so, we used HEpiDISH protocol^36,38^ and a synthetic dataset with samples consisting of nine cell types (Table 4).

**Table 4:** Effect of relative cell proportions on deconvolution accuracy. Deconvolution of a synthetic dataset was performed using HEpiDISH protocol. The synthetic dataset was constructed according to several scenarios where the seven immune cell types summed up to a predefined proportion (from 25 to 80% in increments by 5%). Values are means of three repeats (for each scenario synthetic samples for deconvolution were randomly selected three times).

Parameter of interest		Immune cell proportion not exceeding											
		25%	30%	35%	40%	45%	50%	55%	60%	65%	70%	75%	80%
Spearman correction coefficient between actual and fitted cell proportions	Epi	0.99	0.97	0.99	0.98	0.98	0.97	0.98	0.98	0.99	0.97	0.99	0.99
	Fib	0.99	0.98	0.98	0.97	0.96	0.98	0.99	0.98	0.99	0.98	0.99	0.99
	B	0.47	0.61	0.69	0.91	0.84	0.90	0.92	0.93	0.91	0.96	0.95	0.96
	NK	0.51	0.76	0.65	0.85	0.81	0.89	0.85	0.90	0.93	0.96	0.93	0.94
	CD4T	0.53	0.80	0.81	0.79	0.90	0.90	0.95	0.96	0.94	0.96	0.96	0.98
	CD8T	0.43	0.45	0.57	0.58	0.53	0.80	0.71	0.73	0.87	0.85	0.87	0.90
	Mono	0.94	0.83	0.89	0.87	0.94	0.95	0.98	0.96	0.96	0.98	0.96	0.98
	Neutro	0.81	0.85	0.86	0.90	0.95	0.96	0.97	0.94	0.97	0.96	0.95	0.96
	Eosino	0.56	0.61	0.70	0.71	0.75	0.86	0.93	0.94	0.91	0.92	0.95	0.95
RMSE			0.04	0.04	0.04	0.04	0.04	0.04	0.04	0.04	0.04	0.04	0.04
MedAPE			0.77	0.81	0.78	0.61	0.59	0.61	0.51	0.45	0.45	0.38	0.39

We tested several scenarios where the immune component summed up to a predefined proportion (from 25 to 80% in increments by 5%) and observed that the outcome heavily depended on the relative proportions of different cell types (Table 4). While fibroblasts and epithelial cells estimates were stable across various scenarios (Spearman coefficient never dropping below 0.97), certain immune cell types (specifically CD4T, CD8T, monocytes, neutrophils, eosinophils) were not reliably estimated when present in low amounts. For example, Spearman coefficient was 0.47 indicating an only moderate correlation for B-cells when immune cells were constituting below 25% and reached 0.96 when immune cells summed up to 80%. The latter basically represents a scenario where each of the nine allowed cell types is present in equal proportions (Table 4). RMSE remained stable throughout all tested scenarios, while MedAPE which reflects the accuracy of deconvolution of cells present also in low amounts, dropped from 0.77 (for IC < 25%) to 0.33 (for IC < 80%). This is in line with our prediction that if cell types are present in similar proportions, more precise cell type proportion estimates are possible. Issues related to a reliable estimation of rare cell types have been discussed further (see “Discussion”).

**Figure 3:**
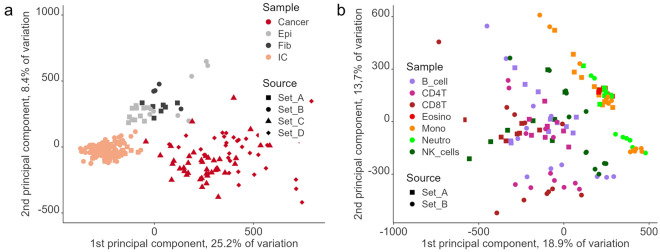
Clustering of various cell types based on methylation profiles. (**a**) four main cell types: cancer cells, epithelial cells, fibroblasts, and immune cells (IC); all immune cell types are presented as a single category, (**b**) immune component zoomed and divided into seven immune cell types. Shape of the data points refers to the source of a particular dataset (Table 2).

The fact that proportions of both epithelial cells and fibroblasts can be reliably estimated irrespectively of their proportions may be caused by them being relatively distant and well separated from immune cells and from each other (Fig. 3a). At the same time, methylation profiles of various immune cell subtypes lie close to one another and therefore devising a deconvolution protocol that would reliably resolve them poses a challenge (Fig. 3b). Of note, samples did not cluster depending on the source of dataset (shape of the datapoints, Fig. 3).

We decided to keep the total proportion of immune cells below 25% when evaluating performance of different deconvolution protocols.

### Modification of the original HEpiDISH protocol

In order to perform a deconvolution of OC samples, it was necessary to include the OC component in our reference panels. The original HEpiDISH does not have this possibility, therefore, we modified the reference panel (see “Materials and methods”), so that it also included the cancer component (set C, Table 2). First, we checked how the original HEpiDISH protocol would classify the ovarian cell lines. As expected, they were mostly recognized as epithelial cells (Supplementary Table S3). Four of them were categorized as samples consisting over 80% fibroblasts (A2780, OVK-18, TOV-112D, TYK-nu), though they were all carcinomas, i.e., originated from epithelial cells. Unlike non-neoplastic cells, which showed resemblance within a cell type and clustered rather tightly, cancer cell lines were loosely scattered and distant from one another (Fig. 3a).

Subsequently, we modified the reference panel used for HEpiDISH so that it also recognized the tumor component. The modified reference panel contained 746 CpGs, allowing separation into the four main types (tumor component, epithelial cells, fibroblasts, and immune cells), and 170 of these CpGs overlapped with the original HEpiDISH. The CpGs used for the second step of deconvolution (prediction of the individual immune cell types) were not modified (see Supplementary Table S1).

As a sanity check, we verified if the HEpiDiSH with the modified reference panel (HEpiDISH-OC) would be able to correctly recognize cell types (including immune cell types) coming from the datasets used for the construction of the original reference panel (Table 2, set A), and from other publicly available datasets (Table 2, set B). Value of one is optimal here and it means that a given sample was recognized as sample consisting in 100% of an underlying cell type. For example, HRPEpiC cell line was classified as 100% epithelium, while HRE as 94% epithelium and 3% fibroblast. On average, HEpiDISH-OC recognized in 92% correctly non-neoplastic samples from its own reference panel (set A) and in 90% correctly samples from the independent dataset (set B; Supplementary Table S4). It also mostly correctly recognized OC cell lines as cancer component. Cell lines included in HEpiDISH-OC reference panel (Table 2, set C) were recognized as samples consisting on average of 97% cancer component. HEpiDISH-OC also performed very well classifying OC cell lines from different sources and not included in its own reference panel (Table 2, set D). Those cell lines were on average recognized as “consisting of 96% cancer component”. For detailed results see Supplementary Table S4.

**Figure 4:**
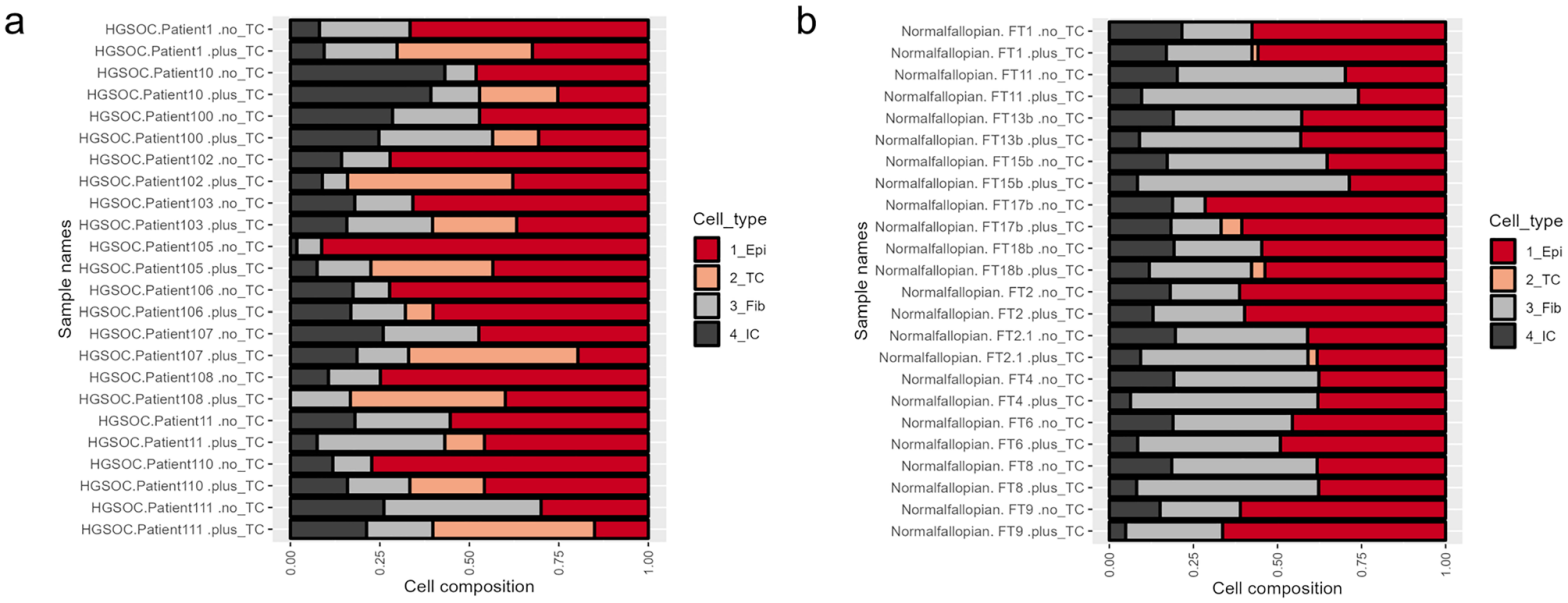
Tumor component (TC) in OC samples comes on the expense of epithelial cell type. Deconvolution of samples from the GSE133556 cohort, performed either with the original HEpiDISH protocol or with HEpiDISH with a modified reference panel (HEpiDISH-OC). (**a**) comparison performed for 12 HGSOC samples (**b**) comparison performed for 12 controls (normal fallopian tissue), demonstrating that the tumor component is largely absent from the control samples.

Then, we ran deconvolution on a set of bulk samples from an available online dataset GSE133556, which includes 99 HGSOC samples and 12 controls^17^ to investigate how HEpiDISH-OC would categorize cancer samples and samples taken from healthy donors, belonging to the same cohort. We compared results of the original (HEpiDiSH) and the modified (HEpiDiSH-OC) protocol and noticed that the tumor component comes almost exclusively from the epithelial cell fraction (Fig. 4). Moreover, cells recognized as tumor cells were largely absent in control (normal fallopian tubes) samples (none detected in 8 out of 12 control samples, overall mean content 1.2% in control samples versus 32% in the tumor samples; Supplementary Table S5).

### Comparison of state-of-the-art methods

To find an optimal deconvolution method for OC samples, we compared performance of the original and modified HEpiDISH with two other state-of-the-art methods: MethylCIBERSORT and ARIC.

In case of MethylCIBERSORT and ARIC, we introduced yet another factor, namely the composition of a reference panel. To check the robustness/sensitivity to the choice of reference datasets we either used (for prediction of the composition of non-neoplastic cells) the samples included in the original HEpiDiSH reference panel (Table 2, set A), an independent set (Table 2, set B) or a combination of both (A and B). The datasets used for predicting the tumor component were either the 43 samples coming from^28^ (Table 2, set C), 40 samples coming from different sources (Table 2, set D) or both sets combined (B and D). Thus, we devised the following methods: (I) HEpiDISH with the original reference panel using set A (this method did not predict proportion of tumor component as a separate category); (II) HEpiDISH-OC with reference panel using sets A for non-neoplastic cells and C for the tumor component; (III) MethylCIBERSORT (MetCIB) with sets A and C; (IV) MetCIB with sets B and D; (V) MetCIB with sets A, B, C and D; (VI) ARIC with sets A and C; (VII) ARIC with sets B and D; VIII) ARIC with sets A, B, C and D. Those methods differed with respect to datasets used for construction of the reference panel, feature selection strategy and the computational algorithm. Supplementary Figure S1 features heatmaps corresponding to each of the eight protocols. These heatmaps illustrate how cell types cluster depending on the algorithm and datasets used to construct a given reference panel.

In order to evaluate performance of all eight methods we used a synthetic dataset (see “Methods”), where the total proportions of all immune cell types did not exceed 25% and where we predicted 10 cell types (including tumor component, in addition to nine allowed in “Defining a range of cell type proportions”). We used the following metrics: Spearman correlation between predicted and known cell proportions, RMSE and MedAPE. Results of performance of all eight methods are gathered in Table 5 as well as the actual and predicted cell content.

**Table 5:** Performance of selected state-of-the-art deconvolution method. A synthetic dataset was used to evaluate the ability of eight methods to correctly predict proportions of ten underlying cell types (apart from the original HEpiDISH protocol, which only predicted nine types). Marked in bold are the results of those three methods which performed best in a given category. If two methods resulted in the same outcome, both were marked in bold (as in case of correlation between actual and predicted by the model proportion of NK cells).

	Ground truth	HEpiDISH	HEpiDISH-OC	MetCIB_AC	MetCIB_BD	MetCIB_ABCD	ARIC_AC	ARIC_BD	ARIC_ABCD
Spearman correlation									
TC	–	0.90	0.95	0.95	0.98	0.96	0.96	0.98	0.98
Epi			0.99	0.98	0.99	0.98	0.99	0.99	0.99
Fib		0.93	0.98	0.98	0.99	0.98	0.98	0.98	0.98
CD4T		0.45	0.44	**0.97**	**0.89**	**0.89**	0.82	0.79	**0.89**
CD8T		NA	NA	0.88	**0.90**	**0.96**	0.87	0.80	**0.94**
B_cell		0.84	0.81	**0.99**	0.97	**0.99**	0.98	0.97	**1.00**
Mono		0.70	0.60	**0.99**	0.98	**0.99**	**0.99**	0.97	**0.99**
NK_cells		0.52	0.58	**0.96**	0.92	**0.98**	**0.96**	0.90	**0.99**
Neutro		0.47	0.48	0.89	0.37	**0.90**	**0.92**	0.35	**0.94**
Eosino		0.74	0.72	**0.95**	NA	**0.98**	**0.95**	NA	**0.99**
Other performance metrics									
RMSE	–	0.040	0.033	**0.027**	0.033	**0.024**	0.028	0.030	**0.024**
MedAPE		0.880	0.675	**0.218**	0.259	**0.202**	0.234	0.241	**0.200**
Content—four main cell types (% total)									
TC	26.2	**53.9**	28.4	25.9	**25.9**	**26.5**	31.6	**26.6**	28.8
Epi	29.0		**29.1**	25.2	30.7	26.8	**29.0**	34.2	32.3
Fib	23.2	**24.4**	**22.2**	24.6	19.8	**22.9**	18.7	19.4	19.0
IC	21.5	**21.7**	**20.3**	24.4	23.6	23.9	**20.7**	19.9	19.9
Content—immune cell types (% total)									
CD4T	2.1	5.2	4.9	**2.0**	1.7	1.5	2.7	**2.1**	**1.8**
CD8T	3.2	0.0	0.0	**3.8**	6.0	5.9	2.0	**3.4**	**3.7**
B_cells	3.6	4.0	**3.8**	**3.6**	4.4	**3.5**	2.8	4.0	3.2
Mono	2.8	3.4	3.2	3.5	3.2	3.2	**3.0**	**2.8**	**2.8**
NK_cells	3.9	5.6	4.9	**4.6**	3.0	**3.1**	**4.3**	**3.1**	2.9
Neutro	2.3	3.3	3.1	**2.9**	5.3	**2.2**	3.0	4.4	**2.2**
Eosino	3.7	0.2	0.2	**3.9**	Not est	4.5	**3.0**	Not est	**3.3**
Times when a given method was among the top-three									
		3	4	**13**	3	**14**	9	5	**14**

Correlation between the proportion of tumor component, fibroblasts and epithelial cells as predicted by all the methods tested and the ground truth was very high. In case of protocols able to predict the content of tumor cells, the Spearman correlation was never observed to drop below 0.95. Correlation coefficients, however, may remain high even if a given method systematically overestimates or underestimates cell proportion. To rule that out, we compared predicted proportions with the ground truth and noticed that only in one case (tumor component estimated by ARIC A + C) the average predicted content differed by more than 5% from the ground truth. Overall, MethylCIBERSORT and ARIC with extended (A + B + C + D-based) reference panels, performed best, regarding the value of Spearman correlations, deconvolution metrics (RMSE, MedAPE) and precision of the estimate (Table 5). At the same time, we could clearly see that when exclusively B + D sets were used for construction of the reference datasets, the performance of both, MethylCIBERSORT and ARIC were worse than when A + C or all 4 sets were used. We speculate that it is due to the fact that B set does not contain eosinophils, and it may affect the overall performance. Of note, in protocols accepting only B + D sets as reference panels, correlation coefficients for all cell types apart from neutrophils was still very high (Table 5).

### Factors affecting performance of deconvolution protocols

Next, we performed deconvolution of the GSE133556 dataset using all seven methods allowing prediction of the tumor component (i.e., we excluded the original HEpiDISH from the analysis). We did so to verify if different methods would result in a similar outcome, but also to see which variables would affect the results. Methods used differed, as mentioned previously, with respect to datasets used for construction of the reference panel, feature selection strategy and the computational algorithm, and we wanted to check which of those variables would affect data clustering.

**Figure 5:**
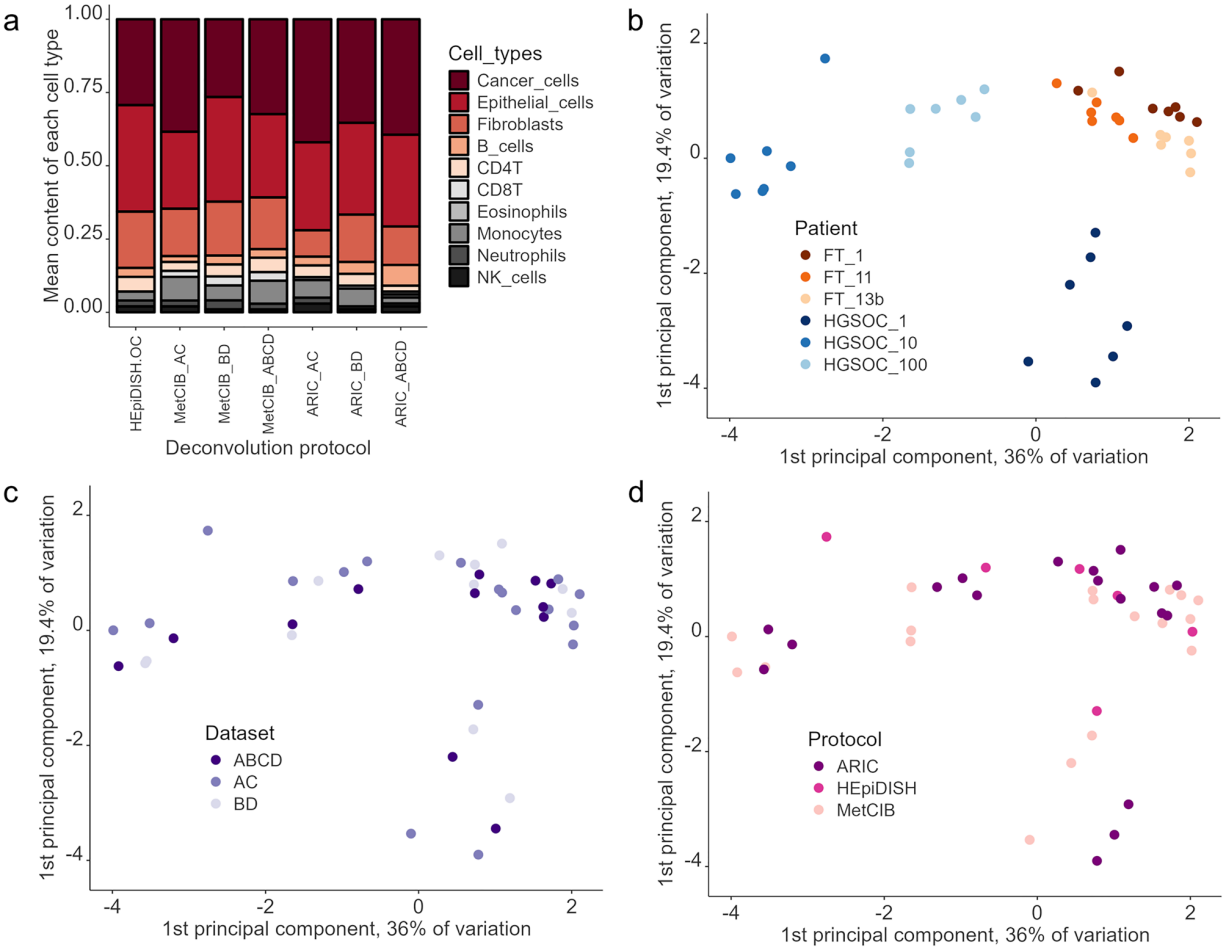
Comparison of seven deconvolution protocols, allowing prediction of the tumor component, for the GSE133556 cohort. (**a**) mean values as predicted by each of the protocols (**b**–**d**) clustering of the deconvolution results for three random HGSOC samples and three random FT controls from the GSE133556 cohort depending on (**b**) patient (**c**) dataset used for constructing of the reference panel (**d**) deconvolution protocol.

All protocols predicted similar mean estimates of the cell type proportions (Fig. 5 and Supplementary Table 6). ARIC A + C overestimated the proportion of tumor component (7% higher than the average for the remaining protocols), which is in line with what we observed for the deconvolution of the synthetic dataset, where ARIC A + C also overestimated the tumor component (Table 5). Again, it was most likely done on the cost of fibroblast fraction. HEpiDISH-OC, again, similarly to the deconvolution of the synthetic dataset, underestimated CD8T cells (Fig. 5a and Supplementary Table S6).

Then, we set to analyze which variables would affect data clustering. We chose randomly three HGSOC samples and three control samples (fallopian tubes from healthy controls) from the GSE133556 cohort and analyzed similarity between cell proportions as predicted by those 7 methods (3 samples × 2 tissue types, i.e., HGSOC or FT × 7 deconvolution methods = 42 datapoints). We expected, that if predictions of the deconvolution protocols are accurate, they should give similar proportions within each sample, and thus datapoints should cluster by the sample or origin. As predicted, HGSOC indeed clustered primarily by the sample of origin, (Fig. 5b–d). For deconvolution data from healthy controls clustering by the sample of origin was less consistent, but still visible (Fig. 5b–d). This may be related to the fact that samples from healthy subjects resemble each other more than HGSOC samples do (lack of tumor fraction, lower content of immune fraction—about 10% compared to 22% in HGSOC cohort as predicted by MethylCIBERSORT). Within samples from the same subject, we did not observe a consistent clustering by algorithm or datasets used for reference panel construction, which can be seen as a positive sign, as the opposite would suggest a systematic bias.

All deconvolution protocols gave comparable results. However, in the following steps we decided to adhere to MethylCIBERSORT with reference panel based on sets A + B + C + D. This decision was motivated as follows. MethylCIBERSORT performed overall better than ARIC and HEpiDISH (Table 5, bottom row). ARIC indeed, estimated the content of immune subtypes more precisely, but was less accurate with the main cell types and tended to overestimate tumor and epithelial content. Importantly, both methods performed better when the extended reference panel (based on all four sets) was used.

### Performance of the optimized method in the study cohort and external datasets

**Figure 6:**
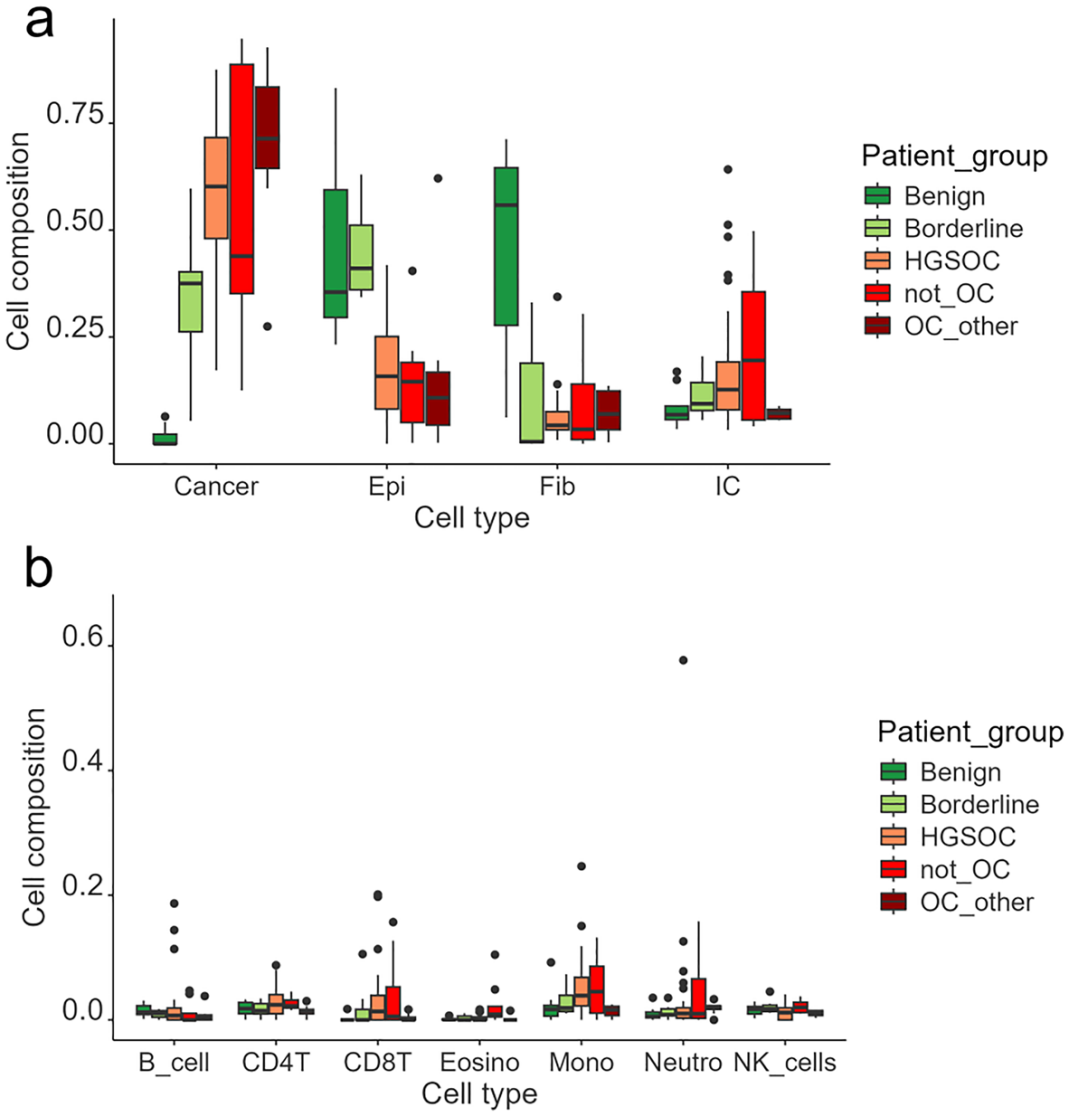
Comparison of sample cell composition across different patient groups in the study cohort. (**a**) main cell groups, namely cancer, epithelial (Epi), fibroblast (Fib), and immune cells (IC), with the latter encompassing all immune cell subtypes into a single category, (**b**) relative proportions of each immune subgroup.

We ran deconvolution of our study cohort using MethylCIBERSORT (Fig. 6 and Supplementary Table 7). As expected, cells recognized as “tumor component” were largely absent in samples from patients diagnosed as “benign”. Interestingly two samples (D7 and G3), both containing marked amounts of tumor component (23% and 35% respectively), were originally diagnosed as benign and only later these patients were re-classified as borderline (Supplementary Table S7). However, the deconvolution protocol recognized these cells as “cancer-like” already in the original samples.

Of note, average proportion of the immune component in all patient groups was predicted to be between 7 and 22%, making the immune cell types the most sparse cell types (Supplementary table 7). It also justified the assumption we made in the first part of our work, where we limited the immune component to 25%, reasoning that the outcome of a deconvolution protocol may be affected by cell types present in low amounts.

We further validated performance of MethylCIBERSORT, with the extended reference panel (based on A, B, C and D sets) in four external cohorts, containing ovarian cancer samples and, in case of two of them, also cancer-free normal controls (Supplementary Table S8). Altogether, 476 cancer samples and 45 normal samples were interrogated. Among the cancer samples, in 17 no cancer component was detected (hence, in 14 serous). Highest tumor content (96.4%) was estimated to be in a HGSOC sample GSM4711707 from cohort GSE155760.

Cancer-like cells were mostly absent from normal tissue samples (none detected in 38 out of 45). Only in four normal samples (two fallopian tube epithelium samples from cohort GSE51820 and two endometrial mucosa from cancer-free normal controls from cohort GSE155760) their content exceeded 10%. Altogether, these results confirm specificity and robustness of the tested method.

## Discussion

When optimizing a deconvolution protocol which could subsequently be used for estimating cell type proportions in OC tissue samples, we assumed that biological as well as computational factors should be taken under consideration. Therefore, we considered both, the choice of datasets used for construction of the reference panel and the choice of a deconvolution protocol. Among methods developed so far, we selected three: HEpiDISH, MethylCIBERSORT and ARIC, which have previously been used in comparative studies and provided reliable cell proportions estimates^37,39^. Methods selected differed with respect to strategy for feature (CpG) selection and the computational algorithm.

One of the challenges we encountered was the need to construct a reliable reference panel. In case of the reference-based deconvolution, if a suitable panel is absent and the study population does not match the reference individuals, for example with respect to age, race, or smoking status, deconvolution results may be biased and unreliable^40^. This issue was relevant also in our case. For example, datasets containing methylation profiles of immune cells from set A did not match our cohort with respect to gender and age (six healthy male blood donors, age 38 ± 13.6 year)^21^. However, the same datasets were included in an extended (containing adipocytes) HEpiDISH reference panel which was later successfully used for deconvolution of breast cancer tissue samples^36^, suggesting that age and gender may not be crucial in this context.

Moreover, information about the epithelial and fibroblast component used to construct reference profiles in our study was based on methylation profiles of established cells lines. For example, information about epithelial cells methylation in HEpiDISH reference panel was based on 11 epithelial cell lines from the ENCODE project, however, none of these cell lines was derived from the ovaries (GSE40699 dataset)^22,36^.

Apart from biological alterations, technical variation, resulting from the fact that reference profiles come from different institutes, may also be a factor. Technical variation may be introduced at several steps, such as bisulfite conversion or CpG methylation array profiling. It can also result from different culturing conditions (in case of cell lines) or from differences in the isolation protocol (in case of immune cells), which may affect sample purity.

Therefore, we reasoned that a reference panel, rather than perfectly matching the study cohort should be as robust as possible. Of note, our study cohort is heterogeneous with respect to age and stage, and, possibly, also to other factors such as for example ethnicity and smoking status. With a broad reference panel, one may catch features characteristic for a given cell type, across biological and technical variation.

Regarding the choice of cell types included in the reference panel, we only allowed seven main immune subtypes, the same as can be found in HEpiDISH^36^. However, we acknowledge that a finer resolution may be desirable. For instance, distinguishing between naïve and memory compartments or between T helper lymphocytes 1 and 2 subtypes as they are respectively related to pro- and anti-inflammatory response, and therefore have distinct biological roles^41^. Deconvolution based on expression profiles already now offers enhanced resolution of immune component. For instance, LM22 reference panel, designed by authors of CIBERSORT, enables the estimation of 22 immune cell types^42^,

Nevertheless, our observations revealed notable discrepancies in the proportions of the seven primary immune cell types predicted by various protocols (refer to Fig. 6). Consequently, we opted to maintain a more granular resolution.

We anticipate that with time, more comprehensive reference methylation panels will emerge. Furthermore, we advocate for independent profiling of each underlying cell type by multiple research groups to accommodate biological and technical disparities. Including tumor component into deconvolution protocol also presents a challenge, since neoplastic transformation may be driven by a plethora of mechanisms and result in genomic disarray, where samples from various patients, or even clonal populations from the same patient, differ greatly.

One approach to solve this problem is to include neoplastic cell lines into reference panels^8,43^. However, including neoplastic cell lines is not ideal, due to possible genomic alterations and cell culture artifacts.

Another option could be using purified tumor cells. Possible challenges it may present would be the necessity to collect a high number of samples to capture inter-patient diversity, proper disaggregation, and purification (to identify and capture cancer cells we need to know their cells markers). Therefore, so far, established cell lines, albeit not being an ideal solution, present a reasonable alternative. Data on significant number of OC cell lines is currently available (Supplementary Table S3 and S4), and since the datasets we implemented come from different projects and different institutes, they present certain biological variation.

We expanded our reference panel, which eventually contained as many as 247 samples, belonging to 10 cell types (Table 2). The payoff is an increased robustness of a reference panel. Whenever possible, we included several different cell lines/samples for the same type of tissue and ensured that the input data came from various sources, i.e., various research units to account for noise resulting from technical variability. In case of most of cell types we managed to obtain stable results.

The notable exceptions were eosinophils and CD8T which content varied across methods used (Supplementary Table S6). However, they were also among the least abundantly represented cell types. Issues with reliable estimation of rare cell types have been previously reported^37,44^.

Other factors which may affect accuracy of a deconvolution protocol are the feature selection strategy and the deconvolution algorithm itself. For instance, while selecting differentially methylated CpGs, HEpiDISH uses one-against-all versus pairwise comparisons in MethylCIBERSORT and ARIC. Moreover, feature selection in HEpiDISH is partially supervised and uses underlying biological knowledge about cell-type specific DNA methylation sites^38^. All three algorithms differ with respect to the underlying computational algorithm (Table 3). Last but not least, HEpiDISH uses a hierarchical deconvolution (resolving immune cell subtypes is a separated step), while MethylCIBERSORT and ARIC are one-step processes^8,36,37^. The role of feature selection and deconvolution algorithm have been systematically assessed by^39^, who demonstrated that most reference-based algorithms yield good results, with one versus all strategy, combined with RPC algorithm being the most robust. The same authors also stated that reference panels build basing either on 450 K or EPIC arrays perform equally well. Since some of the reference profiles were only available as 450 K arrays, and the differentially methylated CpGs had to be present in all underlying datasets, our reference panel was based exclusively on 450 K array^39^.

Since it has been thoroughly investigated previously, in our work, we did not consider each of these factors (feature selection, computational algorithm, one step versus hierarchical deconvolution) separately. When testing performance of selected deconvolution protocols, we mostly applied setting predefined by their respective authors, the way they would be applied by a potential user.

Another factor to consider would be the size of the reference panel, in terms of features (CpGs) included. Our contained around 1200 CpG sites, however, for example Schmidt et al. demonstrated the use of a reference panel containing only 8 CpG sites^45^. One can imagine practical implication of this approach. For example, if a sequencing panel was to be developed for diagnostic purposes, which would at the same time allow correcting for cell composition, it would be reasonable to reduce the number of interrogated CpG sites.

Following the optimization steps, we performed deconvolution of the study cohort as well as altogether five external cohorts (including GSE133556, utilized already during the optimization process). As expected, samples taken from patients diagnosed as benign ovarian cases did not contain or contained low amounts of cells recognized as “cancer-like”, speaking for the accuracy of the deconvolution protocol and the reference panel used. Interestingly though, tumor component was detected in the patient group referred to as “not OC”, i.e., patients where the primary tumor site was not the ovary, but which metastasized to ovaries from distinct locations, even though our reference panel only contained OC methylation profiles. However, cell lines used for its construction, as explained previously, presented a high degree of methylation profiles heterogeneity and did not cluster closely together. Thus, it can be expected that other neoplastic cells, especially of epithelial origin, would present similar degree of disarray and be recognized by an unspecific cancer reference panel. Still, caution should be exercised with this assumption and a reference panel should always be developed for a condition it is going to be used for. Nevertheless, this finding may be beneficial in the real-life scenario where an origin of a neoplastic change manifesting in the ovaries is not always immediately known.

In case of HGSOC cancer samples, the predicted tumor content ranged between 17.2 and 87.6% which may result from tissue sampling and/or nature of the tumor microenvironment. Range of these values speaks for the need to correct for cell heterogeneity in studies on differential gene expression or differential methylation.

## Conclusion

We compared several state-of-the-art methods in order to optimize a robust protocol, suitable for deconvolution of OC tissue samples. We validated the proposed protocol in both, a synthetic dataset, and an external cohort of ovarian cancer samples. When comparing the existing methods, we specifically focused on the role of the reference panel. Subsequently, we tested performance of the selected protocol in our own study cohort, consisting of patients with malignant and benign ovarian disease. This protocol can subsequently be used in exploratory studies or with the perspective to stratify patients’ treatment based for instance on their immune profile. Furthermore, we posit that similar considerations may prove relevant when designing reference panels for other types of cancers.

### Supplementary Information

Supplementary Information 1.

Supplementary Information 2.

Supplementary Information 3.

Supplementary Information 4.

## Data Availability

The datasets (raw data) generated during and/or analyzed during the current study are available from the corresponding author on reasonable request.
