# Supplementary material for: Validating reference-based algorithms to determine cell-type heterogeneity in ovarian cancer DNA methylation studies
Source: Sci Rep. 2024 May 14;14:11048. doi: 10.1038/s41598-024-61857-y (PMC11094148; doi:10.1038/s41598-024-61857-y)
Supplement: Supplementary file 1 — Supplementary Information 1. [file 41598_2024_61857_MOESM1_ESM.zip › Supplementary_Data_Biskup_et_al/Reference_panel_contruction_MethylCIBERSORT_ABCD_markdown_Biskup_et_al.docx]

# loading MethylCIBERSORT package
# remotes::install_github("jysonganan/methylDeConv")
library(MethylCIBERSORT)

# cell types included in the setA
pheno_setA <- c(rep("CD4T", 3), rep("CD8T", 3), rep("B_cell", 3),
 rep("Mono",3), rep("NK_cells", 3),
 rep("CD4T", 3), rep("CD8T", 3), rep("B_cell", 3),
 rep("Mono",3), rep("NK_cells", 3),
 rep("Neutro", 6), rep("Eosino", 6),
 rep("Epi", 11), rep("Fib", 7))
length(pheno_setA)

## [1] 60

# cell types included in the setB
pheno_setB <- c(rep("B_cell", 3), rep("Mono", 3),
 rep(c("Neutro", "CD4T", "CD8T", "NK_cells", "B_cell", "Mono"), 8),
 "Neutro", "NK_cells", "Neutro", "B_cell", "NK_cells", "NK_cells",
 "CD4T", "B_cell", "Neutro", "CD8T", "CD8T", "Mono", "CD4T",
 "CD4T", "Mono", "CD8T", "Neutro", "Mono",
 "CD8T", "Mono", "CD8T", "B_cell", "Mono", "CD8T",
 "CD4T", "B_cell", "CD4T", "Neutro", "B_cell", "NK_cells", "Mono",
 "NK_cells", "B_cell", "CD4T", "Neutro", "CD4T", "NK_cells",
 rep("Epi",8),
 rep("Fib", 5))
length(pheno_setB)

## [1] 104

# combined information about cell types from both sets into object containing information about non-neoplastic cell types
REF_PANEL_NORMAL_A_B.pheno <- c(pheno_setA, pheno_setB)

These objects contain information about the methylation profiles of all the samples and cell lines used for construction of the reference panel (not provided):

load("REF_PANEL_NORMAL_A_B.RData") # 384093 cpg sites in 164 non-neoplastic samples
load("REF_PANEL_OC_LINES_C_D.RData") # 384093 cpg sites in 83 ovarian cancer cell lines)

# This code does the following:
# performs pairwise comparison between cell types to select the most stable features (cpgs)
# saves a reference file to a default location;
# this file should be uploaded as a reference panel (signature) to CIBERSORTx portal
Sig_norm_and_OC_ref_ABCD <- FeatureSelect.V4(CellLines.matrix = REF_PANEL_OC_LINES_C_D,
 Heatmap = FALSE, export = TRUE,
 sigName = "Normal_and_OC_ref_ABCD",
 Stroma.matrix = REF_PANEL_NORMAL_A_B,
 deltaBeta = 0.2, FDR = 0.01, MaxDMRs = 100,
 Phenotype.stroma = REF_PANEL_NORMAL_A_B.pheno)

## Setting up for pairwise feature selection

## Getting Delta Beta estimates

# saving the Sig_norm_and_OC_ref_ABCD object for future use
save(Sig_norm_and_OC_ref_ABCD, file="Sig_norm_and_OC_ref_ABCD.RData")

Sig_norm_and_OC_ref_ABCD is a list object, which is included in the Supplementary Data folder

Sig_norm_and_OC_ref_ABCD.RData includes a dataframe: Sig_norm_and_OC_ref_ABCD$SignatureMatrix of the size 1259 cpg x 247 samples containing information about methylation levels of each sample for each cpg inluded in the panel and is used to limit the deconvolution matrix size before uploading to CIBERSORTx

MAT - is a matrix of beta values to be subjected to deconvolution, for example:

library(GEOquery)

## Warning: pakke 'GEOquery' blev bygget under R version 4.2.1

## Setting options('download.file.method.GEOquery'='auto')

## Setting options('GEOquery.inmemory.gpl'=FALSE)

# load series and platform data from GEO

gset <- getGEO("GSE155760", GSEMatrix =TRUE, getGPL=FALSE)

## Found 1 file(s)

## GSE155760_series_matrix.txt.gz

if (length(gset) > 1) idx <- grep("GPL23976", attr(gset, "names")) else idx <- 1
gset <- gset[[idx]]
head(gset)

## ExpressionSet (storageMode: lockedEnvironment)
## assayData: 6 features, 96 samples
## element names: exprs
## protocolData: none
## phenoData
## sampleNames: GSM4711626 GSM4711627 ... GSM4711725 (96 total)
## varLabels: title geo_accession ... uniqueid:ch1 (51 total)
## varMetadata: labelDescription
## featureData: none
## experimentData: use 'experimentData(object)'
## pubMedIds: 32817081
## Annotation: GPL23976

ex <- exprs(gset)
# log2 transform
qx <- as.numeric(quantile(ex, c(0., 0.25, 0.5, 0.75, 0.99, 1.0), na.rm=T))
LogC <- (qx[5] > 100) ||
 (qx[6]-qx[1] > 50 && qx[2] > 0)
if (LogC) { ex[which(ex <= 0)] <- NaN
ex <- log2(ex) }
dim(ex) # [1] 866238 96

## [1] 866238 96

ex <- na.omit(ex)
dim(ex) # [1] 866238 96

## [1] 866238 96

MAT <- ex

# adjusting the size of MAT to overlap with the reference panel and saves a mixture file
Prep.CancerType(Beta = MAT, Probes = rownames(Sig_norm_and_OC_ref_ABCD$SignatureMatrix), fname = "ExportData_DD_MM_YY")

Mixture file, generated by the code above (ExportData_DD_MM_YY.txt), together with the signature file (Normal_and_OC_ref_ABCD_Signature.txt) generated by the FeatureSelect.V4() function should be uploaded to CIBERSORTx portal
