# Supplementary material for: Validating reference-based algorithms to determine cell-type heterogeneity in ovarian cancer DNA methylation studies
Source: Sci Rep. 2024 May 14;14:11048. doi: 10.1038/s41598-024-61857-y (PMC11094148; doi:10.1038/s41598-024-61857-y)
Supplement: Supplementary file 2 — Supplementary Information 2. [file 41598_2024_61857_MOESM2_ESM.pdf]

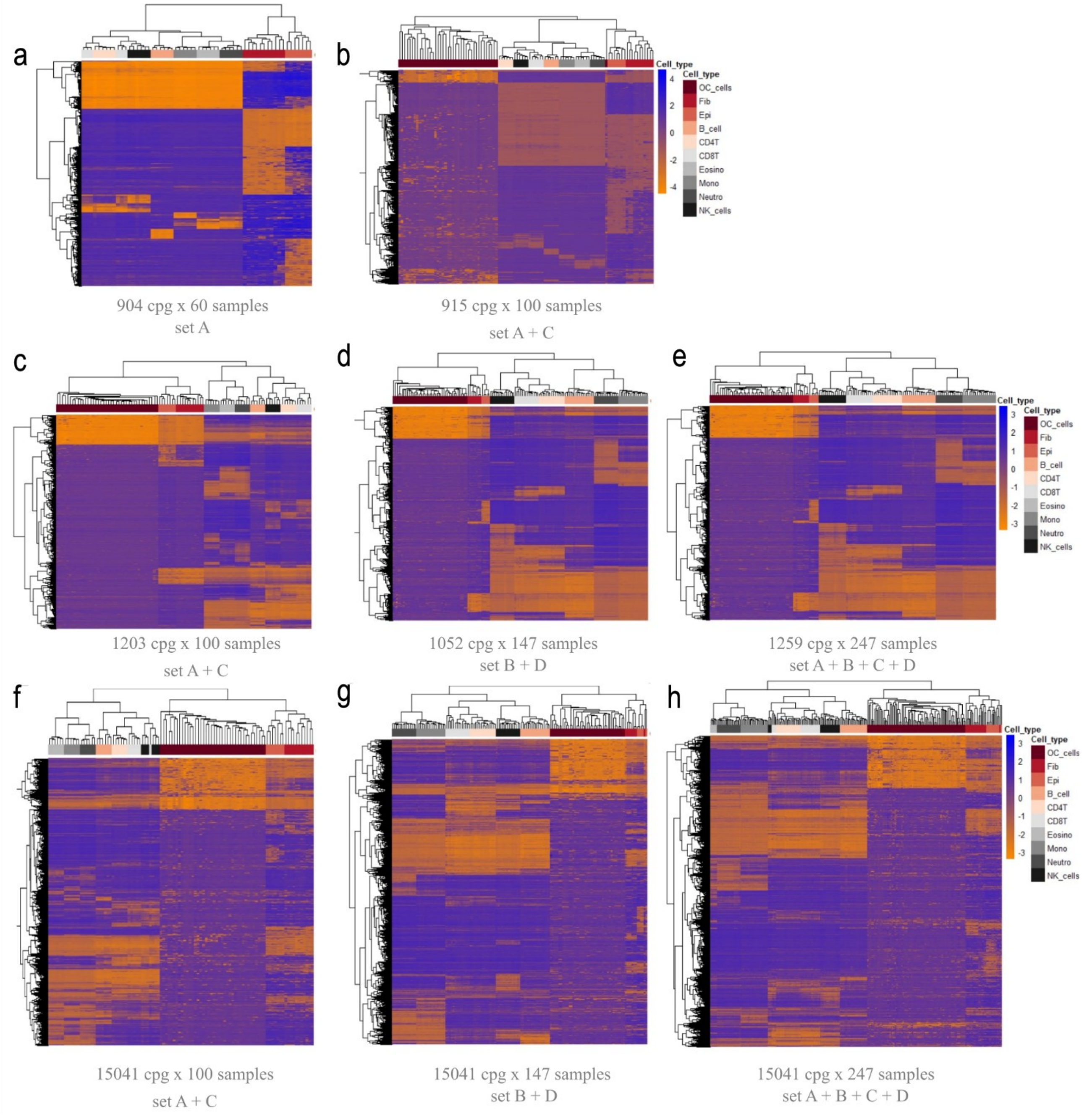

Supplementary fig. S1. Heatmaps illustrating how cell types cluster depending on the algorithm and datasets used to construct a given reference panel. Size of the panel (number of CpG sites x number of samples) and datasets used for its construction are given below each heatmap. In case of ARIC, 15,041 preselected CpGs were used to construct heatmaps. **a)** original HEpiDISH **b)** modified HEpiDISH (HEpiDISH-OC), allowing to estimate tumor component **c-e)** MethylCIBERSORT based on respectively A+C, B+D and A+B+C+D sets **f-h)** ARIC based on respectively A+C, B+D and A+B+C+D sets. Heatmaps were prepared using R software (version 4.2). Input information (number of samples, number of CpG sites and underlying datasets) was generated as described in Suppl. table S1.
