# Supplementary material for: Validating reference-based algorithms to determine cell-type heterogeneity in ovarian cancer DNA methylation studies
Source: Sci Rep. 2024 May 14;14:11048. doi: 10.1038/s41598-024-61857-y (PMC11094148; doi:10.1038/s41598-024-61857-y)
Supplement: Supplementary file 3 — Supplementary Information 3. [file 41598_2024_61857_MOESM3_ESM.docx]

**Validating reference-based algorithms to determine cell-type heterogeneity in ovarian cancer DNA methylation studies**

Edyta Biskup^1^, Joanna Lopacinska-Jørgensen^1^, Lau Kræsing Vestergaard^1^, Estrid Høgdall^1^

^1^ Department of Pathology, Copenhagen University Hospital, Herlev, Denmark

**Correspondence to:** Edyta Biskup; e-mail: [edyta.urszula.biskup-schmoller@regionh.dk](mailto:edyta.urszula.biskup-schmoller@regionh.dk)

**Supplementary table S1**. **Details regarding the usage of each deconvolution protocol**

| **HEpiDISH (original)** | |
| --- | --- |
| **Step 1** | Estimating proportion of the three main cell types (epithelial, fibroblasts and immune cells – all seven types of immune cells as one category)  Reference panel: centEpiFibIC.m (built in the package)  No. of CpGs: 716 |
| **Step 2** | Estimating proportion of the seven immune cell types (B cells, CD4+, CD8+, NK cells, monocytes, neutrophils, eosinophils)  Reference panel: centBloodSub.m (built in the package)  No. of CpGs:188 |
|  | |
| **HEpiDISH-OC (modified version, predicting the proportion of the tumor component)** | |
| **Step 1** | Identifying datasets constituting reference panel in the original HEpiDISH protocol  (see Table 2, set A) |
| **Step 2** | Identifying datasets allowing estimation of the tumor component (see Table 2, set C) |
| **Step 3** | Processing datasets from steps 1 and 2 as described in **Data acquisition and processing of DNAm profiles** and showed in Fig. 1 |
| **Step 4** | Constructing reference panel for estimation of the four main cell types, i.e., epithelial, fibroblasts, immune cells (all seven types as one category) and cancer  Selection criteria: one-against-all, FDR < 0.01, for distance between groups – see **Adding information about tumor component to the HEpiDISH reference panel**  No. Of CpGs: 746 |
| **Step 5** | Constructing reference panel for estimation of the seven immune cell types (B cells, CD4+, CD8+, NK cells, monocytes, neutrophils, eosinophils)  Selection criteria: no new selection criteria; the centBloodSub.m reference panel (built in the package) was kept  No. of CpGs: 169 out of the original 188 due to the filtration applied to datasets constituting reference panel (set A, Table 2) as described in **Data acquisition and processing of DNAm profiles** and showed in Fig. 1  NB: values in the reference panel are median beta values for a given cell type |
| **Step 6** | Estimating proportion of the four main cell types, i.e., epithelial, fibroblasts, immune cells (all seven types as one category) and cancer  Reference panel: see step 4 |
| **Step 7** | Estimating proportion of the seven immune cell types (B cells, CD4+, CD8+, NK cells, monocytes, neutrophils, eosinophils)  Reference panel: see step 5 |
|  | |
| **MethylCIBERSORT** | |
| **Step 1** | Identifying datasets to be included in reference panels  For non-neoplastic cells:  set A (Table 2): in total 60 samples from 9 cell types; these datasets are the same as in the original and modified HEpiDISH protocols  set B (Table 2): in total 104 samples, 8 cell types, these datasets do not overlap with the reference panel used in HEpiDISH protocols; Table 2  For the tumor component:  set C (Table 2): in total 43 samples; these datasets are the same as datasets used to identify tumor component in HEpiDISH-OC  set D (Table 2): in total 40 samples; these datasets do not overlap with datasets used to identify tumor component in HEpiDISH-OC |
| **Step 2** | Processing datasets from step 1 as described in **Data acquisition and processing of DNAm profiles** and showed in Fig. 1 |
| **Step 3** | Grouping datasets in order to construct different reference panels:  A + C – contains datasets from sets A and C, Table 2 (ten cell types: nine normal and tumor component)  B + D – contains datasets from sets B and D, (Table 2; nine cell types: eight normal – no eosinophils - and tumor component)  A + B + C + D – contains datasets from sets A, B, C and D (Table 2; ten cell types: nine normal and tumor component) |
| **Step 4** | Preparation of three reference panels, for each of the combinations from step 3, using function FeatureSelect.V4 (built in MethylCIBERSORT package)  Selection criteria: pairwise comparison between cell types, FDR > 0.01, distance between groups (deltaBeta) = 0.2, max number of features per comparison = 100  No. of CpGs for A+C reference panel: 1,203  No. of CpGs for B+D reference panel: 1,052  No. of CpGs for A+B+C+D reference panel: 1,259  NB: values in the reference panel are median beta values (as percent, not a fraction of 1) for a given cell type |
| **Step 5** | Preparations of the dataset to be deconvolved, using function Prep.CancerType (build in MethylCIBERSORT package), separately for each reference panel.  Prep.CancerType function filters out probes from the dataset which do not appear in the reference panel and changes the format of beta values (from a fraction of 1 to percentage) |
| **Step 6** | Deconvolution using CIBERSORTx platform, separately for each reference panel  Number of iterations: 500 |
|  | |
| **ARIC** | |
| **Step 1** | Identifying datasets to be included in reference panels  Same as MethylCIBERSORT (see MehylCIBERSORT, step 1) |
| **Step 2** | Processing datasets from step 1 as described in **Data acquisition and processing of DNAm profiles** and showed in Fig. 1 |
| **Step 3** | Grouping datasets in order to construct different reference panels:  Same as MethylCIBERSORT (see MehylCIBERSORT, step 3) |
| **Step 4** | Preselection of features (CpG sites) to be used as input for ARIC.  Comment: Preselection is necessary as using complete methylations profiles would be too computationally heavy for the program to run the algorithm. CpGs which differ between cell types were identified using FeatureSelect.V4 function (built in MethylCIBERSORT package), however, using less strict criteria than in MethylCIBERSORT itself.  Input: sets A+C, B+D and A+B+C+D  Tool: function FeatureSelect.V4 (built in MethylCIBERSORT package)  Selection criteria: pairwise comparison between cell types, FDR < 0.01, distance between groups (deltaBeta) = 0.2, max number of features per comparison = 1,000  No. of preselected CpGs for A+C: 10,715  No. of preselected CpGs for B+D: 9,866  No. of. preselected CpGs for A+B+C+D: 11,261  Final no. of CpGs: 15,404 (due to partial overlaps, the number of preselected CpGs is not a sum of CpGs obtained for ich combination of datasets) |
| **Step 5** | Constructing of three preliminary reference panels (A+C, B+D, A+B+C+D), so that each contains only 15,404 CpGs preselected in Step 4  NB: values in the reference panels are median beta values for a given cell type |
